# What Will the Harvest Be?

**Published:** 1900-08

**Authors:** 


### Editorial.

### WHAT WILL THE HARVEST BE?

THE traveller along the dusty highways at this period of the year can enjoy the landscape with its variety of color, and the undulations of grain-covered fields with the heavily laden heads, indicating a hopeful outlook for a bountiful harvest. The statistician and the gambler in the wheat-pit each can closely figure upon the world's great annual harvest, and each in his way can tell us how many billions of bushels of wheat will be added to the world's granaries for the coming year.

At this period, also, another harvest has been ingathered. It is not in wheat or corn, but in graduated men and women, the harvest of intelligence that is sent forth yearly to enlighten the world and to feed other minds, and thus the cycle of a world's life moves on, the physical and mental correlating each to the other and adding to the sum total of human knowledge and experience.

The record is made up for the year in all dental colleges. Hundreds of young men and women have passed the crucial tests of college and State Board examinations, and are now scattered far and wide seeking a resting-place somewhere to work out the problem of life.

When the thought centres upon this great addition to the army of dental workers, the query naturally arises, What will the harvest be? In other words, will this large addition work to the good or ill of the dental profession in this country? In a former article there was an effort made to prove that the number of graduates, great as it is yearly, was not in excess of the growth of population or increase in intelligence among the laity in regard to teeth. This view is still held, and while some are discouraged when they read the long lists of graduates, there is apparently no reason for this pessimistic view. When the interests of the individual are considered, there may be cause for anxiety.

The college man, the teacher, regards this annual harvest with some feelings of mental disturbance. He cannot look at it from the point of view of the practitioner or statistician, but combining these feelings he adds, in his view, the most important query, Has the harvest been productive of increased perfection? Has the work of the year developed a higher class of dentists? Will those who have graduated in the colleges the present spring and summer mean for the dental profession a higher standard of practical excellence? The mental examination he gives himself is not encouraging, and he is forced into the conviction that this higher standard has not been attained, and cannot be under present methods.

The fault, if it exists, does not lie with the teaching, or, perhaps, with the plans adopted, but with the time allotted the undergraduate. In the years past and, happily, gone two years were considered quite sufficient to make a doctor of medicine or a doctor of dentistry. Gradually this has been changed until four years is regarded as the least possible time for the former and three years for the latter. The rapid growth in all branches of medical study, and this necessarily includes dentistry, will make it impossible to limit the time of study to the periods named. Medicine must go to five years, and eventually dentistry must follow.

To those who have been educated under old methods and have been measurably successful in practice, such continual lengthening of the terms may seem not only foolish but suicidal. These, however, while undoubtedly sincere in their convictions, lack the experience necessary to form correct opinions. The additions made to the curricula of all the colleges, especially dental, give a too limited period to accomplish the work desired. This has been heretofore fully considered on these pages, but its importance does not seem to have been impressed on dental educators to any perceptible degree.

The reason for this does not lie in the necessity for an increase in time, for this is certainly clear to all, but to a feeling that the schools are not financially equal to bearing the additional burden. In other words, a four-year course will mean the ultimate extinction of many of the dental schools of this country and the crippling of all. While this may be in part true, this selfish side of the question should not have weight. Many of our dental schools are not up to the highest standard, indeed, are essentially inferior in all that goes to make up a dental college. Their departure from the stage of activity would not be a serious blow to the world's progress, yet the loss of all honest effort in this direction is to be deplored. The temporary crippling of the higher schools means to them eventually a broader foundation and a higher intellectual and practical life.

While this is being written the delegates from distant localities are on their way to Old Point Comfort to meet with the organization known as the National Association of Dental Faculties. When this article appears it will then be known to our readers whether this Association was prepared to reach the high level outlined here, a full four years' course, or that the members decided foolishly to continue the shorter term, thus simply deferring for a brief space the inevitable. Whether this be the result or not, there is no cause for discouragement. The trend of civilization is forever onward, notwithstanding there are times, like the present, with its wars and its barbarisms, when it seems tending to a lower level. So with college work,—a step in advance, a step backward; but as the years roll on there is a steady advance to the goal,—the ideal of the true educator.

The harvest may not be all that could be desired at the present period of educational work, but the indications all point to a future when all antagonizing forces will be brought into harmonious relations and an educational equilibrium be established.

